# Pulse–Glide Behavior in Emerging Mixed Traffic Flow Under Sensor Accuracy Variations: An Energy-Safety Perspective

**DOI:** 10.3390/s25134189

**Published:** 2025-07-05

**Authors:** Mengyuan Huang, Jinjun Sun, Honggang Li, Qiqi Miao

**Affiliations:** 1 School of Automotive and Transportation Engineering, Shenzhen Polytechnic University, Shenzhen 518055, China; huangmy2215@szpu.edu.cn; 2 School of Civil Engineering and Transportation, Northeast Forestry University, Harbin 150040, China; jinjunsun2001@nefu.edu.cn; 3 College of Mechatronic and Electrical Engineering, Northeast Forestry University, Harbin 150040, China; 4 School of Economics and Management, Dalian University of Technology, Dalian 116024, China; miaoqiqia1@163.com

**Keywords:** emerging mixed traffic flow, connected and automated vehicle, pulse and glide, energy conservation, traffic safety, sensor accuracy

## Abstract

Pulse and Glide (PnG), as a fuel-saving technique, has primarily been applied to manual transmission vehicles. So, its effectiveness when integrated with a novel vehicle type like connected and automated vehicles (CAVs) remains largely unexplored. On the other hand, CAVs have evidently received less attention regarding energy conservation, and their prominent perception capabilities clearly exhibit individual variations. In light of this, this study investigates the impacts of PnG combined with CAVs on energy conservation and safety within the emerging mixed traffic flow composed of CAVs with varying sensing accuracies. The results indicate the following: (i) compared to the traditional driving modes, the PnG can achieve a maximum fuel-saving rate of 39.53% at Fuel Consumption with Idle (FCI), reducing conflicts by approximately 30% on average; (ii) CAVs, equipped with sensors boasting a greater detection range, markedly enhance safety during vehicle operation and contribute to a more uniform distribution of individual fuel consumption; (iii) PnG modes with moderate acceleration, such as 1–2 m/s^2^, can achieve excellent fuel consumption while ensuring safety and may even slightly enhance the operational efficiency of the intersection. The findings could provide a theoretical reference for the transition of transportation systems toward sustainability.

## 1. Introduction

With the rapid development of the economy, communication technologies represented by 5G have been continuously advanced, and computational capabilities driven by chip development have significantly improved, leading to substantial progress in communication and automation technologies [1,2]. Consequently, connected and automated vehicles (CAVs), which integrate advanced communication and automation technologies, have emerged. Timely and stable communication, coupled with rapid and precise automated control, positions CAVs as a promising solution to enhance traffic safety, provide more convenient travel options, and optimize traffic efficiency. Moreover, CAVs can operate in platoons, existing in traffic flows in a novel form [3,4]. Specifically, at the automation level, the environmental perception capabilities enabled by Vehicle-to-Vehicle (V2V) and Vehicle-to-Infrastructure (V2I) communications make it highly likely for CAVs to improve vehicle safety [5,6]. Safety assessments of CAVs, conducted through sensitivity analysis or the establishment of safety evaluation frameworks, indicate that CAVs can theoretically reduce conflicts and effectively enhance traffic safety [7,8]. Furthermore, through V2V and V2I communications, CAVs can optimize intersection operational efficiency by ensuring safety through path planning within intersections [9,10]. On the other hand, CAV platooning can achieve smaller inter-vehicle gaps, thereby improving road utilization and impacting traffic capacity. However, the safety and stability during and after platoon formation still require further exploration, particularly in addressing potential communication-related issues [11,12].

Additionally, the advent of CAVs presents opportunities for optimizing vehicle energy efficiency [13,14]. For instance, Feng et al. [15] conducted path planning for CAVs with fuel consumption as the optimization objective, coordinating with intersection signals through communication capabilities, resulting in a 13.8% reduction in exhaust emissions compared to vehicles without such planning and coordination, thereby reducing fuel consumption. However, with increased vehicle usage and shifts in travel patterns, the positive environmental impacts of CAVs may be offset [16,17]. Therefore, a comprehensive examination of CAVs’ environmental impacts, particularly regarding fuel consumption, is both meaningful and necessary to fully explore their applications.

In the energy sector, the situation regarding resource depletion and global warming remains critical. Transportation accounts for approximately 20% of global primary energy consumption and about 18% (~5.3 Gt) of total anthropogenic greenhouse gas emissions, with passenger cars contributing a significant proportion [18]. Consequently, reducing fuel consumption is an indispensable objective for the future development of vehicles, transportation systems, and society at large. Effective methods to reduce vehicle fuel consumption include reducing vehicle weight, improving tire rolling resistance, optimizing aerodynamic drag, enhancing drivetrain systems, and increasing engine efficiency [19]. However, vehicle operation is significantly influenced by driver behavior [20,21], which can affect fuel consumption, as manifested through vehicle trajectories and speeds [22,23].

Compared to trajectory control, speed control is more precise and direct. Pulse and Glide (PnG), which involves alternating between pulsing and gliding deceleration to cycle between high and low speeds to achieve energy conservation [24], stands out due to its operational simplicity and practical advantages [25,26]. For instance, Salgueiredo et al. [27] conducted real-vehicle experiments and simulations on a straight test track, confirming the energy conservation effectiveness of PnG through its operational mechanism. Furthermore, the PnG strategy has demonstrated energy conservation potential on roads with gradients, in simple car-following scenarios where a following vehicle trails a lead vehicle and in automated vehicle platoons traveling on single-lane roads [28,29]. However, it is evident that PnG studies are constrained by strict assumptions, and the integration benefits from CAVs, a novel type of non-manually controlled vehicle, remain unclear, particularly in more realistic and complex traffic environments.

In summary, this study employs simulation methods to examine the fuel consumption and safety impacts of PnG within the emerging mixed traffic flow composed of different types of CAVs at the intersection. The research is conducted in three key aspects: (i) modeling based on the real-world intersection and designing three types of CAVs based on variations in vehicle sensor accuracy for the emerging mixed traffic flow; (ii) establishing different safety constraints for the following vehicle based on the states of the ego and leading vehicles, and selecting distinct driving modes according to the type-specific attributes of CAVs; (iii) controlling the acceleration during the pulsing phase of PnG mode, formulating various PnG modes, and using the constant speed (CS) mode as a baseline to analyze the fuel consumption and safety impacts of each PnG mode based on measures such as Hourly Fuel Consumption (HFC), Fuel Consumption with Idle (FCI), and Rate of Conflict (RC).

The structure of the remaining sections of this paper is as follows: Section 2 provides a review of the relevant research on CAVs and PnG. Then, Section 3 describes the development of the simulation model, the composition of the emerging mixed traffic flow, the safety constraints and control strategies for vehicles, and the evaluation measures employed. Subsequently, Section 4 presents the results of the simulation runs and their subsequent processing. Following this, Section 5 analyzes the fuel consumption and safety impact of different vehicle types under various PnG modes based on the results. Finally, Section 6 summarizes the differences in vehicle fuel consumption and safety caused by varying PnG modes and vehicle perception capabilities, identifies the most suitable PnG mode for adoption, and highlights the limitations of this study as well as directions for future research.

## 2. Literature Review

With the emergence of CAVs, Human-Driven Vehicles (HDVs) will coexist with CAVs for an extended period, leading to a transition from homogeneous traffic flows to mixed traffic flows, despite variations in the Market Penetration Rate (MPR) of CAVs [30,31]. In such scenarios, ensuring safety between CAVs and HDVs is naturally a primary concern [32,33]. As the MPR of CAVs increases, safety within mixed traffic flows can be enhanced [34,35]. Moreover, when the MPR of CAVs falls within a certain range, their ability to form platoons can increase road traffic capacity while maintaining high safety levels [36,37]. Regarding the MPR of CAVs, it is evident that for the foreseeable future, the MPR will remain relatively low, influenced not only by technological advancements but also by public acceptance and economic conditions [38,39].

On the other hand, CAV-related technologies are subject to iterative development, particularly in terms of autonomous driving capabilities, where different CAVs exhibit varying levels of automation [40,41]. Additionally, the proportion of CAVs with different automation levels within the overall CAV population is undoubtedly varied, with lower automation levels offering significant advantages in terms of cost and utilization rates [42,43]. As a critical component supporting CAVs’ automation capabilities, sensors collect data on the vehicle’s state and surrounding environment, providing environmental perception capabilities [44,45,46]. It is well established that, regardless of their automation level, all CAVs rely on sensors for support. However, sensor performance varies across CAVs, particularly in terms of detection range and accuracy [47,48]. Therefore, in scenarios with low MPR of CAVs, it becomes imperative to investigate the potential impacts of emerging mixed traffic flows composed of CAVs with varying perception capabilities on safety and other aspects.

Undoubtedly, in terms of energy conservation, CAVs can partly achieve reductions in vehicle fuel consumption through methods such as motion control or coordination with traffic management systems, although this aspect has received relatively limited attention [49,50]. Among these, planning the speed and travel routes of CAVs to achieve energy conservation represents a feasible approach [51,52]. The PnG, which inherently involves speed planning, stands out due to its simplicity in planning and the high implementability of speed control, making its application to CAVs for energy conservation highly promising. For instance, Li et al. [53] applied PnG on a straight single-lane highway to a single autonomous vehicle, a manually driven vehicle following an autonomous lead vehicle, and a mixed traffic flow consisting of 30 autonomous and manually driven vehicles. Through numerical simulations, they found that, compared to CS driving, PnG effectively improves fuel economy, with the presence of autonomous vehicles amplifying this effect. Clearly, the application scenarios of PnG, particularly when integrated with autonomous vehicles or even CAVs, are subject to strict constraints, as vehicle control strategies in traffic flows must also account for the states of surrounding vehicles. Therefore, in emerging mixed traffic flows that consider objective conditions such as sensor accuracy, constraining the safety of CAVs and exploring the safety and fuel consumption impacts of combining PnG with CAVs hold significant practical importance.

## 3. Methods

### 3.1. Construction of Road Model

**Figure 1:**
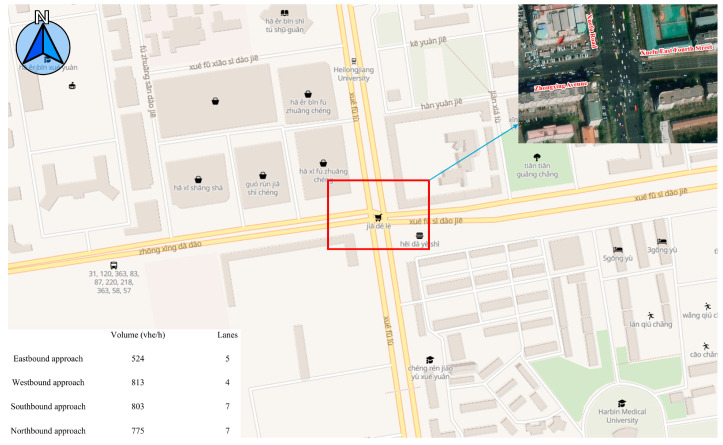
Information on the target intersection.

Due to the structural complexity of intersections and their critical role in urban traffic management systems, they are highly representative of the urban traffic flow. In light of this, this study utilizes the intersection as a platform to investigate the impacts of PnG combined with CAVs on urban traffic from the perspectives of fuel consumption and safety. Specifically, a traffic survey was conducted at the intersection of Xuefu Road, Zhongxing Avenue, and Xuefu East Fourth Street in Nangang District, Harbin City, to obtain data on road geometry, signal timing, and traffic flow, which served as the foundation for simulation modeling. Evidently, this is not a fully regular intersection, with its specific location details presented as shown in Figure 1. And the data collection and processing methods are detailed in Lu et al. [54] (note: the data were used with authorization from the authors of [54]).

### 3.2. Design of the Emerging Mixed Traffic Flow

Cameras, LiDARs, and radars are the primary sensors employed by CAVs for environmental perception [55]. Among these, cameras have a maximum detection range of no more than 20 m, yet they remain the predominant sensor type in certain vehicle models. The detection range of LiDARs varies depending on the manufacturer and model, encompassing ranges within 50 m, within 100 m, and exceeding 100 m. Radars, in turn, can be categorized into long-range radars (10–250 m), medium-range radars (1–100 m), and short-range radars (0.15–30 m), with their configuration primarily determined by the vehicle’s equipped advanced driver-assistance systems [56]. Given that the current autonomous driving level of CAVs is predominantly at L1 or lower, the manufacturers, models, and costs of sensors adopted across different vehicle types vary significantly and lack standardization, with sensor accuracy also exhibiting differences. Additionally, the shortest approach lane length at the target intersection is approximately 130 m. Considering these factors collectively, three distinct types of CAVs were defined: CAV-S7, CAV-S2, and CAV-S1.

Among these, CAV-S7 possesses a forward target detection capability of 50 m, with an MPR set at 70%. CAV-S2 is capable of detecting targets within a 100 m range ahead, with an MPR of 20%. In contrast, CAV-S1 has a detection range of 130 m and an MPR of 10%. Additionally, for all CAV types, a random fluctuation in detection accuracy ranging from 85% to 100% is permitted. Against this backdrop, CAV-S7, CAV-S2, and CAV-S1 collectively constitute the emerging mixed traffic flow proposed in this study.

### 3.3. Control Strategy for Vehicles

#### 3.3.1. Car-Following Model

Given that CAVs possess V2V communication capabilities, cooperative adaptive cruise control (CACC) is typically employed as the car-following model [57]. This enables CAVs to autonomously maintain a predefined time gap with the leading vehicle while facilitating faster and earlier responses to traffic events. In this study, the CACC model proposed by Milanes et al. [58] is adopted as the car-following model for CAVs. Its core component is a spacing-regulation-based controller, the structure of which can be expressed as follows:

(1) $$X_{i}\left(s\right)=G\left(s\right)U_{i}\left(s\right)$$

where $X_{i}\left(s\right)$ denote the position of vehicle in the queue; $G\left(s\right)$ is the vehicle dynamic model; and $U_{i}\left(s\right)$ represents the target speed command for vehicle.

Here, assuming that the vehicle starts from a stationary state, the relationship between the ego vehicle and leading vehicle can be expressed as:

(2) $$X_{i}\left(s\right)=\frac{D\left(s\right)+G\left(s\right)K_{P}\left(s\right)}{1+G\left(s\right)\left[K_{P}\left(s\right)P_{P}\left(s\right)+K_{L}\left(s\right)P_{L}\left(s\right)\right]}X_{i-1}\left(s\right)$$

where $D\left(s\right)$ represents the delay in wireless communication; $K_{P}\left(s\right)$ and $K_{L}\left(s\right)$ denote the time interval error adjustment controllers for the following vehicle and leading vehicle, respectively; $P_{P}\left(s\right)$ and $P_{L}\left(s\right)$ represent the car-following strategies for the following vehicle and leading vehicle, respectively.

Among them, the specific forms of controllers $K_{P}\left(s\right)$ and $K_{L}\left(s\right)$ are:

(3) $$K_{P}\left(s\right)=k_{1}s+k_{2}$$

(4) $$K_{L}\left(s\right)=k_{3}s+k_{4}$$

where $k_{1}$ and $k_{2}$ denote the control gain for following vehicle; $k_{3}$ and $k_{4}$ are the control gain for leading vehicle.

The specific forms of car-following strategies $P_{P}\left(s\right)$ and $P_{L}\left(s\right)$ are:

(5) $$P_{P}\left(s\right)=h_{P}s+1$$

(6) $$P_{L}\left(s\right)=h_{L}s+1$$

where $h_{P}$ and $h_{L}$ denote the target values of time headway for following vehicle and leading vehicle.

The vehicle dynamic model is a second-order transfer function, and its specific form is as follows:

(7) $$G\left(s\right)=\frac{k}{s^{2}+2\theta \omega_{n}s+\omega_{n}^{2}}e^{-T_{d}s}$$

where k, θ, $\omega_{n}$ and $T_{d}$ represent static gain, damping coefficient, natural frequency and time delay, respectively.

#### 3.3.2. Planning for Speed

Given the targeted nature of the PnG mode and the CS mode in terms of velocity, ensuring safety during mode activation is imperative [59]. To this end, this study designates the presence of a leading vehicle as the primary criterion for mode selection. In the absence of a leading vehicle, if the global activation switch for the PnG mode is disabled, the vehicle defaults to the CS mode. Under these conditions, CAV-S7 is permitted to travel at a constant speed of 30 km/h, CAV-S2 is allowed to maintain a constant speed of 40 km/h, and CAV-S1, with the most advanced perception capability, can operate at a constant speed of 50 km/h. Conversely, the vehicles will adopt the PnG mode. Specifically, CAV-S7 operates in PnG mode with an average speed of 30 km/h and a speed fluctuation range of ±5 km/h. CAV-S2 employs a PnG mode with an average speed of 40 km/h, also with a ±5 km/h fluctuation range. Meanwhile, CAV-S1 utilizes a PnG mode with an average speed of 50 km/h, maintaining the same ±5 km/h speed fluctuation range. Notably, due to the significant differences in speed between CS mode and PnG mode, separating the control modules of these two modes based on the status of the global activation switch for the PnG mode allows for more precise monitoring of the activation status of the PnG mode as well as determining whether the vehicle is in the pulsing or gliding phase.

In contrast, when a leading vehicle is present, the primary objective of the vehicle is to ensure safety. Given that the target intersection is signalized, queuing and waiting scenarios are common. Safety constraints must be established based on the speed state of the leading vehicle. Specifically, when the leading vehicle’s speed is 0, the distance between the following vehicle and the leading vehicle must satisfy the requirements for emergency braking, given the vehicle’s braking capability. Conversely, when the leading vehicle’s speed is non-zero, if the following vehicle’s speed exceeds that of the leading vehicle, the Time to Collision (TTC) must be greater than 2 to ensure safety and prevent rear-end collisions [60]. The calculation formula for TTC is as follows:

(8) $$TTC=\frac{HD}{v_{f}-v_{l}}$$

where $v_{l}$ denotes the speed of leading vehicle, m/s; $v_{f}$ represents the speed of following vehicle, m/s; HD is the headway between following vehicle and leading vehicle, m.

The mathematical expressions for the safety constraints under different conditions are presented in Equation (9).

(9) $$\left\{\begin{array}{cc} \frac{v_{f}{\left(t\right)}^{2}}{2a}-HD-L_{v}>0 & v_{l}=0\\ TTC>2 & v_{l}\neq 0 \end{array}\right.$$

where $L_{v}$ denotes the length of the vehicle, m, the value in this study being 4.5; a is the maximum deceleration of the vehicle, m/s^2^.

After satisfying the aforementioned safety constraints, vehicles must still determine their driving strategy based on the global activation switch. When the PnG mode is not enabled, i.e., when the constant speed mode is activated, CAV-S7 is permitted to operate at a constant speed of 30 km/h. In contrast, CAV-S2 and CAV-S1 need to dynamically adjust their driving strategies based on the distance to the leading vehicle. Specifically, when a CAV-S2 detects a leading vehicle and the inter-vehicle distance is less than 50 m, it adopts a constant speed mode of 30 km/h. However, if the distance exceeds 50 m, the vehicle operates at a constant speed of 40 km/h. For a CAV-S1, when the distance to the leading vehicle is less than 50 m, it is similarly restricted to a constant speed of 30 km/h. If the distance falls within the 50–100 m range, it operates at a constant speed of 40 km/h. However, when the distance exceeds 100 m, CAV-S1 engages in a constant speed mode with an average speed of 50 km/h.

On the other hand, to further explore the impacts of the PnG mode, with CAVs as the platform, on urban transportation, this study starts from its operational mechanism and designates the acceleration during the pulsing phase as the control variable. As is widely known, the maximum acceleration of conventional passenger vehicles typically reaches around 5 m/s^2^, though accelerations of 3 m/s^2^ or higher are generally considered extreme. To determine the choice of acceleration, a survey was conducted to gather public preferences regarding the acceptable acceleration levels during the pulsing phase of the PnG mode. The results revealed no significant bias in public preference toward either very low accelerations (e.g., within 1 m/s^2^) or extreme accelerations (e.g., 3 m/s^2^ or above). In light of this, to refine the analysis of the first phase of the PnG mode, which is also the fuel-intensive phase, and to assess the impact of acceleration, this study considered an acceleration threshold ranging from 0 to 3.5 m/s^2^ with 0.5 m/s^2^ intervals, thereby encompassing both normal and extreme conditions. A total of seven distinct PnG modes were designed accordingly. The specific vehicle control mode schemes are presented in Table 1.

#### 3.3.3. Measures for Fuel Consumption and Safety

To comprehensively evaluate the fuel consumption and safety impacts of different vehicle control strategies, this study examines vehicle fuel consumption from both temporal and spatial perspectives. Additionally, the RC and the Waiting Time per Vehicle (WTV) are adopted as measures to analyze safety conditions. Specifically, the fuel consumption measures include the Total Fuel Consumption (TFC) generated within the intersection, the HFC for vehicles passing through the intersection, the Fuel Consumption per Vehicle (FCV) at the intersection, the average fuel consumption at the intersection including FCI, and the average fuel consumption at the intersection excluding idling fuel consumption (FC).

First, TFC statistically calculates the fuel consumption of all vehicles during the simulation operation, and its mathematical expression is:

(10) $$TFC=\frac{\sum_{i=1}^{n}f_{i}\left(t\right)}{720000}$$

where $f_{i}\left(t\right)$ denotes the instantaneous fuel consumption of the i-th vehicle at time t, mg; n represents the total number of vehicles; and the gasoline density used here is 0.72 g/mL, so 1 L of gasoline is equivalent to 720,000 mg.

And the HFC focuses on the instantaneous fuel consumption of vehicles while operating within the intersection, which can be expressed as:

(11) $$HFC=\frac{TFC\times 3600}{t}$$

where t is the total operation time of vehicles, s.

FCV, on the other hand, represents the allocation of TFC to each vehicle, with its specific form expressed as:

(12) $$FCV=\frac{TFC}{C_{v}}$$

where $C_{v}$ denotes the number of vehicles during the simulation.

FCI extrapolates the fuel consumption performance of vehicles within the intersection to a 100 km journey, with its calculation method presented in Equation (13) as follows:

(13) $$FCI=\frac{TFC\times 1000\times 100}{td}$$

where td represents the total mileage of all vehicles, m.

Considering the control of right of way at signalized intersections, FC excludes idling fuel consumption and then extrapolates the result to a 100 km journey, with its expression given as:

(14) $$FC=\frac{\left(TFC-F_{w}\right)\times 1000\times 100}{td}$$

where $F_{w}$ denotes the fuel consumption during waiting time, L.

It should be noted that, in the fuel consumption analysis, when vehicles are in the gliding phase of the PnG mode, this study adopts an engine fuel-cutoff approach, recording the fuel consumption at that moment as 0.

On the other hand, RC is defined as the ratio of the number of instances where the TTC is less than 1.5 s to the number of instances where the ego vehicle’s speed exceeds that of the leading vehicle and can be expressed as:

(15) $$RC=\frac{PC}{TTC_{d}}$$

where PC denotes the number of vehicles that may be in conflict; $TTC_{d}$ represents the number of vehicles whose TTC is less than 1.5 s [61].

## 4. Results

Upon completing the model development, parameter configuration, and operational testing, the simulation can be formally executed to generate output, with the resulting data specifically presented in Table 2. Evidently, in terms of fuel consumption and safety, the introduction of the PnG mode, compared to the CS mode, effectively enhances energy efficiency and ensures safety.

**Figure 2:**
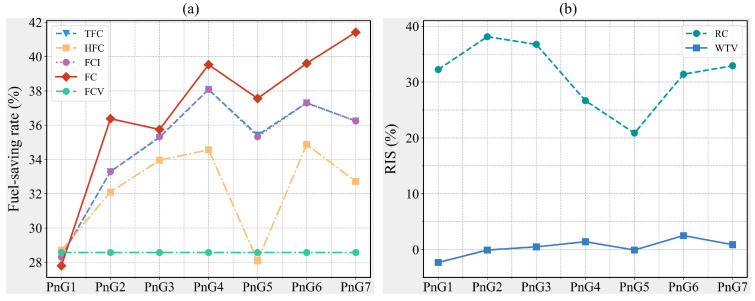
Changes in fuel consumption and safety. (**a**) Fuel consumption under different control strategies. (**b**) Safety under different control strategies.

After further processing of the raw data, it can be observed from Figure 2a that, compared to the CS mode, the PnG mode achieves comprehensive improvements in fuel consumption. The fuel-saving rate refers to the percentage of fuel consumption saved in the PnG mode compared to the CS mode, with its calculation process detailed in Equation (16) as follows:

(16) $$fsr=\frac{f_{png}-f_{cs}}{f_{cs}}\times 100$$

where fsr is the fuel-saving rate; $f_{png}$ denotes the fuel consumption of PnG mode, mg; and $f_{cs}$ represents the fuel consumption of CS mode, mg.

And the variation in safety is represented by the Rate of Improvement for Safety (RIS), which is mathematically defined as follows:

(17) $$RIS=\frac{V_{CS}-V_{P}}{V_{CS}}\times 100$$

where $V_{CS}$ is the values of measures for safety under CS mode; $V_{P}$ denotes the values of measures for safety under PnG mode

Notably, in terms of FC, the minimum fuel-saving rate exceeds 25%, with the maximum value approaching 42%. Regarding safety, as indicated by the directly related safety measure, RC, the PnG mode effectively reduces vehicle conflicts, with an average improvement in RC of approximately 30%, as shown in Figure 2b. For the indirectly related safety measure, WTV, the PnG mode generally has minimal impact, and in certain PnG modes, it even achieves reductions. This suggests that the PnG mode has little to no adverse effect on the operational efficiency within the intersection, avoiding congestion that could compromise safety.

## 5. Discussion

### 5.1. Fuel Consumption Under Different Driving Modes

**Figure 3:**
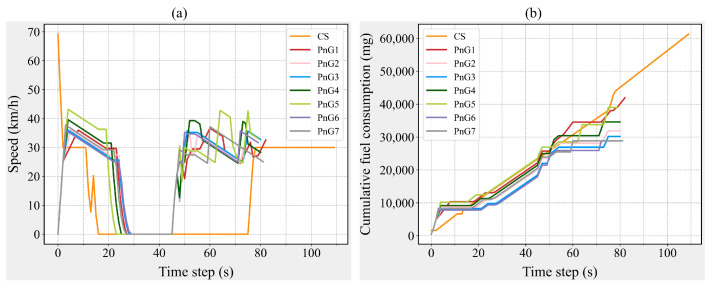
Speed and cumulative fuel consumption of a CAV-S7. (**a**) Speed variations of a CAV-S7. (**b**) Cumulative fuel consumption changes of a CAV-S7.

From the perspective of individual vehicles, traversing the intersection in PnG mode effectively reduces fuel consumption for this segment of the journey, with the impact of acceleration changes being minimal, as the FCV across all PnG modes remains consistent. However, from the perspective of other fuel consumption measures, an increase in acceleration does not uniformly enhance energy efficiency. Notably, the PnG1 mode ranks last in performance across all measures, as illustrated in Figure 2a. Given that CAV-S7 has an MPR as high as 70% and constitutes the primary component of the traffic flow, the fuel consumption variation in CAV-S7 is likely the main contributor to this phenomenon. During the simulation, the speed and instantaneous fuel consumption of each vehicle are recorded at every time step. By randomly selecting a CAV-S7 and analyzing its speed and fuel consumption patterns, the impact of different control modes on the operation of CAV-S7 can be investigated from a more microscopic perspective (see Figure 3). As shown in Figure 3a, the acceleration duration in the PnG1 mode is significantly longer and more frequent than in other PnG modes, resulting in a higher fuel consumption increment compared to other PnG modes. Nevertheless, due to the fuel savings during the gliding phase, the cumulative fuel consumption of the PnG1 mode remains lower than that of the CS mode, as depicted in Figure 3b.

Additionally, Figure 2a reveals that the PnG5 mode exhibits lower values for TFC, HFC, FCI, and FC compared to the PnG4 and PnG6 modes, positioning it at the trough of the fuel consumption curve. Evidently, the gliding duration in the PnG5 mode is shorter than in the PnG4 and PnG6 modes, which significantly reduces the fuel savings achievable through gliding. Moreover, an acceleration of 2–2.5 m/s^2^, likely within the vehicle’s acceleration performance threshold, allows it to readily reach a higher peak speed in the next time step, thereby increasing instantaneous fuel consumption over the distance. Consequently, the combination of reduced gliding duration and a higher attainable peak speed results in the PnG5 mode exhibiting higher fuel consumption than the PnG4 and PnG6 modes.

On the other hand, the acceleration profiles of the PnG4, PnG6, and PnG7 modes can easily satisfy the designated speed fluctuation range, enabling rapid attainment of the target speed and subsequent gliding. This allows these modes to glide for longer durations and reduce waiting time at the approach, leading to superior performance in TFC, HFC, FCI, and FC measures.

**Figure 4:**
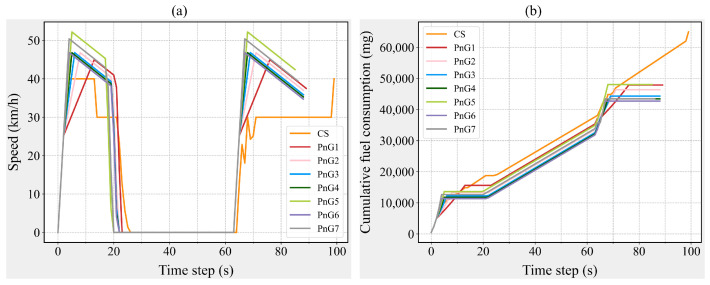
Speed and cumulative fuel consumption of a CAV-S2. (**a**) Speed variations of a CAV-S2. (**b**) Cumulative fuel consumption changes of a CAV-S2.

Subsequently, a CAV-S2 is randomly selected, and its speed and instantaneous fuel consumption data are extracted, as shown in Figure 4. Similar to the situation with CAV-S7, for CAV-S2, the PnG1 mode also exhibits the longest acceleration duration, resulting in a correspondingly shorter gliding duration. Meanwhile, compared to other PnG modes, the PnG5 mode still achieves the highest peak speed, leading to substantial instantaneous fuel consumption, as shown in Figure 4. Consequently, for CAV-S2, the fuel consumption of the PnG1 and PnG5 modes remains elevated. On the other hand, due to CAV-S2’s greater detection range compared to CAV-S7, it can adopt PnG modes with a higher average speed when both distance and safety conditions are met. The speed ceiling of 45 km/h aligns well with acceleration rates of 1.5 m/s^2^, 2.0 m/s^2^, and 3.0 m/s^2^. Therefore, for CAV-S2, the PnG3, PnG4, and PnG6 modes demonstrate commendable fuel consumption performance.

**Figure 5:**
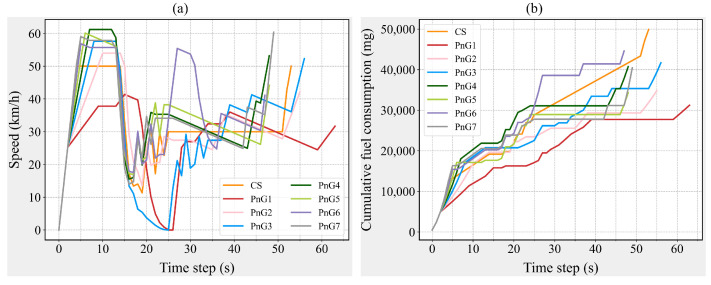
Speed and cumulative fuel consumption of a CAV-S1. (**a**) Speed variations of a CAV-S1. (**b**) Cumulative fuel consumption of a CAV-S1.

Similarly, a CAV-S1 is randomly selected as the subject of observation, and its speed and fuel consumption data are extracted, as shown in Figure 5. For the CAV-S1, its fuel consumption performance under the PnG1 mode was notably superior, primarily due to its shorter acceleration duration and longer gliding phase, as illustrated in Figure 5. It is well known that larger acceleration fluctuations tend to result in higher instantaneous fuel consumption. When different PnG modes are required to reach the same peak speed, the PnG1 mode typically exhibits a prolonged acceleration phase due to its relatively low acceleration, which may lead to cumulative fuel consumption during this phase that exceeds that of other PnG modes. However, in this case, CAV-S1 appears to have detected a closely leading vehicle during the PnG1 operation, thereby limiting its peak speed to 35 km/h, well below the 45 km/h upper limit typically associated with PnG modes. The combination of a shorter acceleration period, lower instantaneous fuel consumption resulting from reduced acceleration, and the longest gliding duration contributed to the vehicle’s excellent energy performance under PnG1.

On the other hand, owing to CAV-S1’s advanced perception capabilities, it must dynamically adjust its speed based on the distance to the leading vehicle, while ensuring safety. Consequently, its fuel consumption performance under different PnG modes can vary significantly depending on its specific driving state, as shown in Figure 5b.

**Figure 6:**
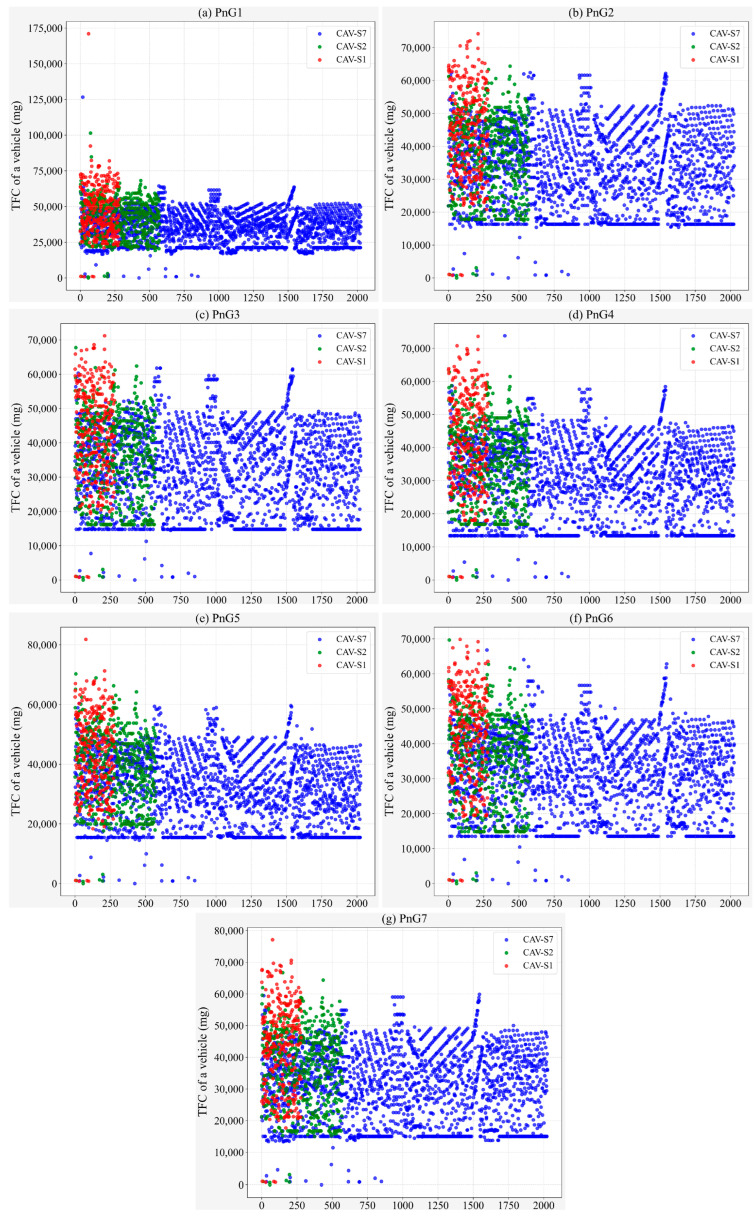
Fuel consumption of vehicles under different PnG modes with different sensors.

**Figure 7:**
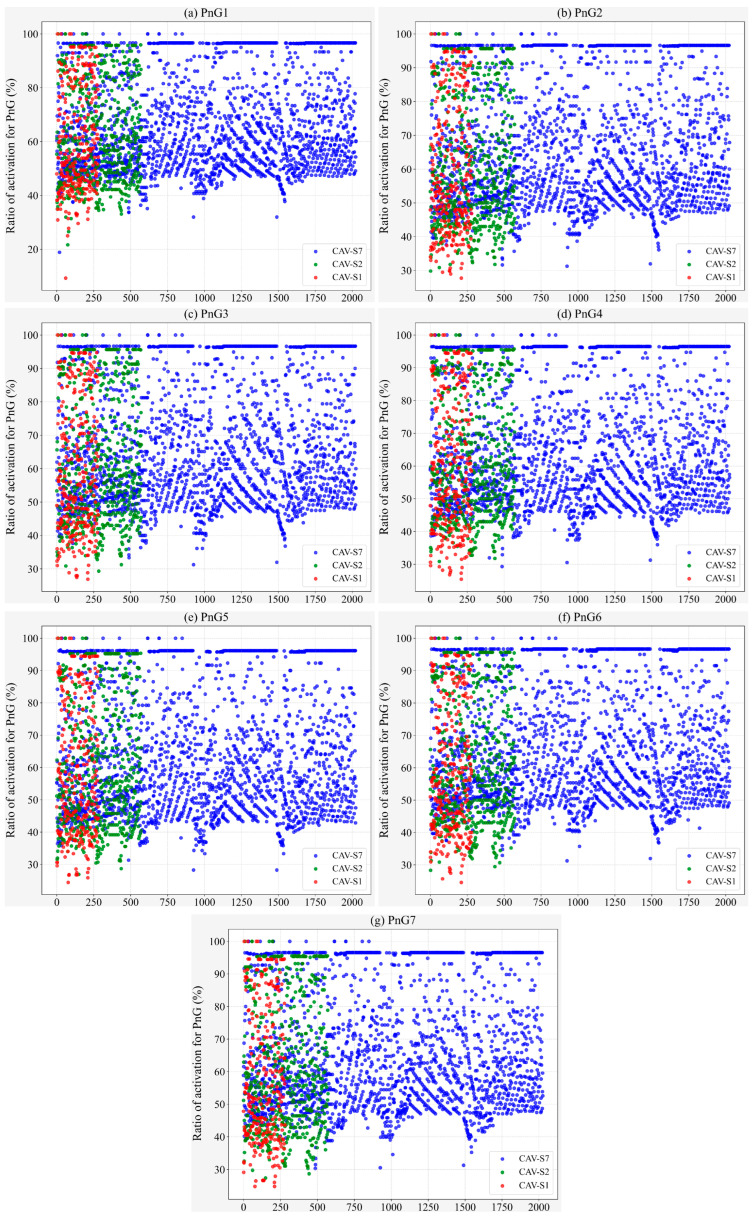
Ratio of activation for different PnG modes with different sensors.

**Figure 8:**
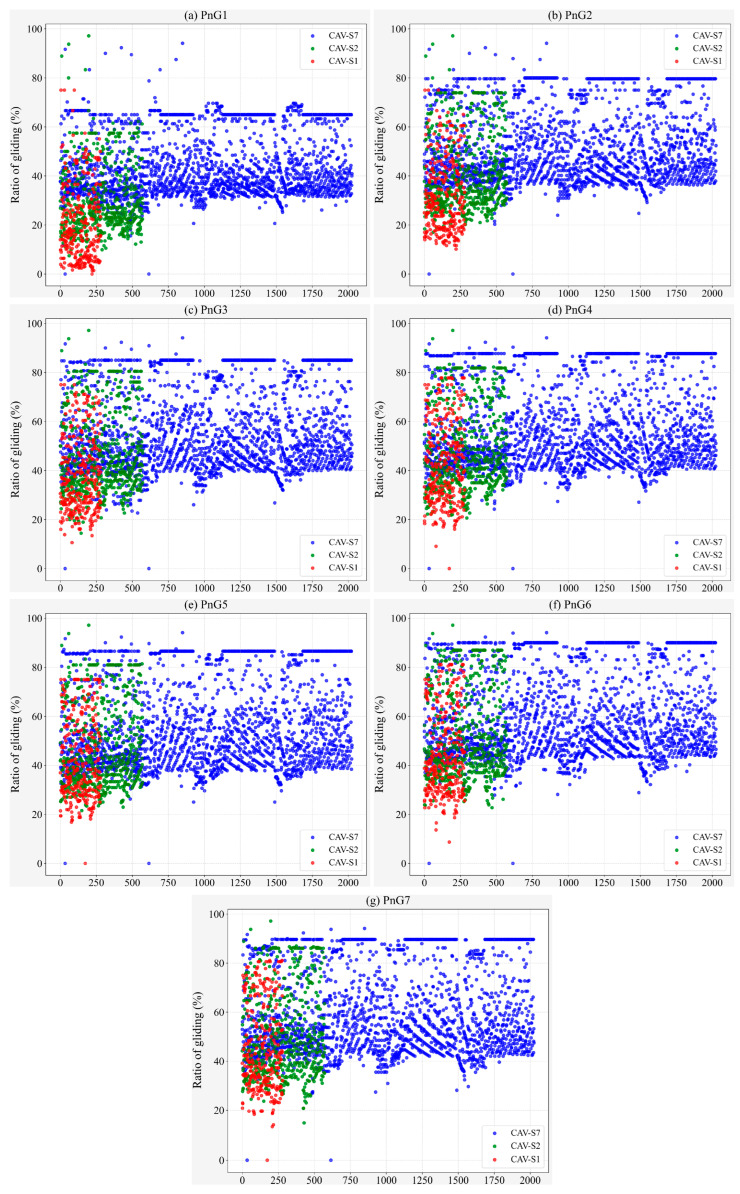
Ratio of gliding for different PnG modes with different sensors.

On the other hand, from the perspective of traffic flow, the TFC conditions of different types of CAVs under different control modes are observed, as shown in Figure 6. Firstly, it is obvious that in the PnG1 mode, fuel consumption is more concentrated (Figure 6a). In this mode, CAV-S7 emerges as the primary contributor to intersection fuel consumption, with its TFC falling within a range of 5000–25,000 mg. Moreover, the activation rate of the PnG1 mode for CAV-S7 is lower compared to other modes, predominantly within the 40–60% interval (Figure 7a). Additionally, vehicles operating in the PnG1 mode exhibit a lower gliding activation rate than in other modes, which significantly undermines the energy conservation potential of this mode (Figure 8a).

Evidently, gliding is the critical factor for PnG mode to achieve its energy conservation effect. However, for the vehicle to enter the gliding phase, it must first ensure that the PnG mode is activated. As illustrated in Figure 7, compared to CAV-S7, both CAV-S2 and CAV-S1 have a longer detection range, resulting in a higher proportion of vehicles with an increased PnG activation rate. Furthermore, as the acceleration level of the PnG mode increases, the activation rate distribution on CAV-S1 becomes more uniform (Figure 7).

Furthermore, the gliding time steps of vehicles with activated PnG mode are extracted to obtain the proportion of gliding time during vehicle operation, as shown in Figure 8. Compared to CAV-S7 and CAV-S2, CAV-S1 does not exhibit a significant advantage in terms of gliding rate, indicating that CAV-S1 spends most of its operational time in the pulsing phase. Due to its ability to reach higher peak speeds, CAV-S1 is subject to more stringent safety constraints and must frequently adjust its speed, which ultimately results in higher overall fuel consumption compared to CAV-S7.

### 5.2. Safety Under Different Driving Modes

As shown in Figure 2b, the PnG1 mode leads to a slight increase in WTV, primarily due to the extended acceleration duration required for CAV-S7 to reach its peak speed under this mode. This prolonged pulsing phase results in a relatively high overall travel time. Nevertheless, since the PnG mode repeatedly executes acceleration maneuvers whenever activation conditions are met, it is still capable of reducing the overall travel time compared to the CS mode.

**Figure 9:**
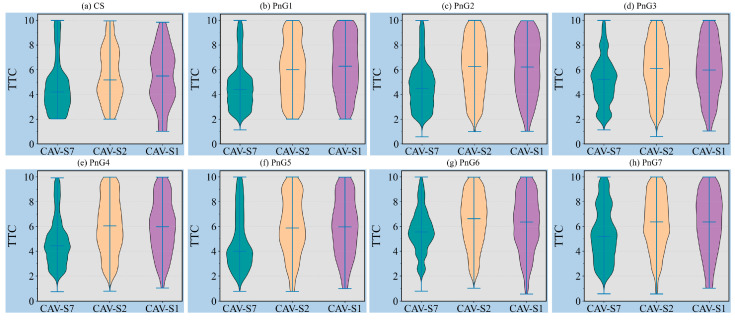
Distribution of TTC under different control modes with different sensors.

Evidently, autonomous vehicles, equipped with perception and control capabilities, can accurately select and implement control strategies based on their real-time operational state while adhering to predefined safety constraints. When a leading vehicle is present and the following vehicle’s speed exceeds that of the leading vehicle, the TTC can intuitively reflect the level of conflict between the two vehicles, thereby enabling an assessment of their safety, as shown in Figure 9. As shown in Figure 9a, even under the CS mode, CAVs are able to maintain safety, with the traffic flow’s TTC predominantly concentrated in a range of 2 to 6 s. Furthermore, as indicated in Table 2, the conflict rate is only 13.09%, effectively minimizing the risk of collisions [62].

On the other hand, Figure 9 demonstrates that CAV-S2 and CAV-S1, equipped with longer detection ranges, are capable of identifying leading vehicles earlier and selecting more diverse control strategies based on inter-vehicle distance and relative speed. As a result, their TTC values are significantly higher than those of CAV-S7. Additionally, with increasing acceleration levels in the PnG modes, the TTC of CAV-S7 generally increases. However, it exhibits a decline in PnG4 and PnG5, followed by a rebound in PnG6 and PnG7. This trend aligns with the observations in Figure 2b and can be largely attributed to the high MPR of CAV-S7.

Moreover, compared to the CS mode, the TTC values under PnG modes are largely concentrated in safer ranges. Specifically, in the PnG1 mode, most TTC values exceed 3 s, while in other PnG modes, the majority of TTC values are even greater than 4 s.

## 6. Conclusions

This study aimed to investigate the fuel consumption and safety implications of Pulse and Glide (PnG) modes within a novel mixed traffic flow consisting of connected and automated vehicles (CAVs) with varying perception capabilities, in urban traffic scenarios. To this end, an intersection model was constructed based on real-world traffic flow data. Three types of CAVs, including CAV-S7, CAV-S2, and CAV-S1, were designed to reflect differences in sensor detection ranges, each equipped with distinct Market Penetration Rates (MPRs) and perception errors. Vehicle speeds were planned based on their driving states, using Time to Collision (TTC) and other indicators as safety constraints. Taking the acceleration level during the PnG pulsing phase as a control variable, and using Fuel Consumption without Idle (FC), Rate of Conflict (RC), and Waiting Time per Vehicle (WTV), and so on, as the measures, this study explored how different PnG modes affect not only fuel consumption and safety but also overall traffic efficiency.

The results indicate that, with the support of CAV technologies, various PnG modes can achieve both significant fuel savings and safety assurance. The maximum fuel-saving rate in terms of TFC reaches up to 39.53%. Even under the constant speed (CS) mode, the RC at intersections is as low as 13.09%. When enhanced by PnG modes, this rate can be further reduced by approximately 30% on average, with the lowest observed RC reaching 8.10%, thereby demonstrating a clear improvement in traffic safety. Regarding WTV, when the acceleration level in the PnG mode is too low, it leads to prolonged acceleration durations, which may slightly hinder intersection operational efficiency. However, this does not compromise traffic safety. Moreover, moderate acceleration levels (e.g., 1–2 m/s^2^) can actually optimize WTV while simultaneously achieving favorable fuel consumption outcomes and maintaining high safety standards. On the other hand, compared to CAV-S7, both CAV-S2 and CAV-S1 exhibit more advanced perception capabilities. The TTC values observed in these vehicles are generally higher than those in CAV-S7, with many values clustering above 6 s. This clearly supports enhanced operational safety at the vehicle level as well as improved safety across the overall traffic flow.

Overall, these findings affirm the energy conservation potential and safety of PnG + CAV in urban transportation. Based on this, we propose that automakers could integrate the PnG mode into CAVs in the future. When the average vehicle speed is low, an acceleration of 1 m/s^2^ can be employed for the PnG mode, whereas at higher average speeds, an acceleration of 2 m/s^2^ may be adopted. Furthermore, signal timing at intersections can be coordinated based on the proportion of vehicles utilizing the PnG mode within the traffic flow, thereby optimizing energy consumption at the intersection while maintaining operational efficiency. However, further consideration is warranted regarding the impact of factors such as the speed fluctuation range of PnG and the MPR of CAVs on their fuel-saving effects. Additionally, the energy conservation potential under varying traffic flow conditions merits further investigation. It is noteworthy that these conclusions are derived from the specific methodologies and simulations employed in this study. Future research could explore adjusting the activation proportion of the PnG mode based on drivers’ driving styles to further enhance its energy conservation potential.

## Figures and Tables

**Table 1:** Parameters for speed under different control modes.

Parameters	CS	PnG1	PnG2	PnG3	PnG4	PnG5	PnG6	PnG7
Acceleration (m/s^2^)	0	0.5	1.0	1.5	2.0	2.5	3.0	3.5
Deceleration (m/s^2^)	0	0.16	0.16	0.16	0.16	0.16	0.16	0.16

**Table 2:** Results of fuel consumption and safety.

Measures	CS	PnG1	PnG2	PnG3	PnG4	PnG5	PnG6	PnG7
TFC (L)	210.16	150.60	140.20	136.01	130.12	135.72	131.77	133.99
HFC (L/h)	3.24	2.31	2.20	2.14	2.12	2.33	2.11	2.18
FCV (L/veh)	0.07	0.05	0.05	0.05	0.05	0.05	0.05	0.05
FCI (L/100 km)	15.23	10.92	10.16	9.85	9.43	9.85	9.55	9.71
FC (L/100 km)	12.70	9.17	8.08	8.16	7.68	7.93	7.67	7.44
RC (%)	13.09	8.87	8.10	8.28	9.60	10.36	8.98	8.78
WTV (s)	22.82	23.35	22.84	22.71	22.50	22.84	22.25	22.62

## Data Availability

Data are only available upon request due to restrictions regarding, e.g., privacy and ethics. The data presented in this study are available from the corresponding author upon request. The data are not publicly available due to their relation to other ongoing research.
